# Use of CeO_2_ Nanoparticles to Enhance UV-Shielding of Transparent Regenerated Cellulose Films

**DOI:** 10.3390/polym11030458

**Published:** 2019-03-11

**Authors:** Wei Wang, Baikai Zhang, Shuai Jiang, Huiyu Bai, Shengwen Zhang

**Affiliations:** Key Laboratory of Synthetic and Biological Colloids, Ministry of Education, School of Chemical and Material Engineering, Jiangnan University, Wuxi 214122, China; zbkjnu@163.com (B.Z.); 15606185233@163.com (S.J.); bhy.chem@163.com (H.B.); shengwen_zh0825@163.com (S.Z.)

**Keywords:** CeO_2_ nanoparticles, regenerated cellulose films, UV shielding, hydrophobicity

## Abstract

The major challenge in preparing polymer nanocomposites is to prevent the agglomeration of inorganic nanoparticles (NPs). Here, with regenerated cellulose (RC) films as supporting medium, UV-shielding and transparent nanocomposite films with hydrophobicity were fabricated by in situ synthesis of CeO_2_ NPs. Facilitated through the interaction between organic and inorganic components revealed by X-ray diffraction (XRD) and Fourier transformation infrared spectroscopy (FTIR) characterization, it was found that CeO_2_ NPs were uniformly dispersed in and immobilized by a cellulose matrix. However some agglomeration of CeO_2_ NPs occurred at higher precursor concentrations. These results suggest that the morphology and particle size of CeO_2_ and the corresponding performance of the resulting films are affected by the porous RC films and the concentrations of Ce(NO_3_)_3_·6H_2_O solutions. The optimized nanocomposite film containing 2.95 wt% CeO_2_ NPs had more than 75% light transmittance (550 nm), high UV shielding properties, and a certain hydrophobicity.

## 1. Introduction

Nanocomposites of polymers and inorganic nanoparticles (INPs) have attracted increasing interest due to their value-added applications derived from their unique optoelectrical, magnetic, electrical, thermal, and antibacterial properties [1,2,3,4]. However it is difficult to maintain a good dispersion and nanoscale stability of the INPs in the polymer matrix, due to the aggregation of the INPs [4]. Among the various polymeric materials, biopolymers are considered as environmentally friendly and sustainable materials with versatile functionalities which could meet the requirements for numerous applications [5]. Recently cellulose materials were reported as an ideal platform for the design and preparation of advanced inorganic-polymer hybrid materials, either as template, support or precursor [6,7], because this most important natural polymer, with a special hierarchical order of supramolecular structure, possesses distinguishing properties such as hydrophilicity, strong mechanical properties, high flexibility, as well as significant adsorption and swelling behavior, resulting in the possibility for potential application in various advanced materials [8].

In general, three ways are applicable to prepare inorganic-cellulose nanocomposites. (i) By mixing of the suspensions of nanocelluloses (CNs) and INPs, nanocomposites based on CNs can be prepared. CNs usually need to be surface modified to promote the dispersion of INPs in the cellulose matrix [9]. (ii) By incorporating INPs into the dissolved cellulose matrix and then regenerating, nanocomposites based on regenerated cellulose (RC) are available [10]. Based on porous cellulose fibers, new applicable and facile techniques can be designed for preparing functional materials, however, good dispersion and chemical stability of the INPs in the corresponding cellulose solvents are required. (iii) Most surprisingly, with an RC gel or wet film and cellulose fabrics as template or support, benefiting from the abundant surface hydroxyl groups and the high microscopic porosity of the cellulose fibers, the uniform dispersion of INPs such as plate-like Fe_2_O_3_, silver, and Co_3_O_4_ NPs, in the cellulose matrix could be achieved expediently by in-situ synthesis [11,12,13]. However, the synthesis conditions of INPs are limited, and the porous structure of the cellulose substrate greatly affects the size and morphology of the nanoparticles.

As a colorless polysaccharide, cellulose-based materials facilitate the fabrication of transparent products. Cellulosic materials with photo-functionality such as UV absorption, optoelectrical, light-diffusing, photoluminescent, and visible light-induced photocatalytic properties have been prepared by introducing different functional INPs such as metallic NPs and quantum dots [9,14,15,16,17]. Among various photofunctional INPs, cerium dioxide (CeO_2_) is considered to be a better ultraviolet radiation absorbent. Due to a relatively small band gap (3.1 eV), compared to TiO_2_ (3.27 eV) and ZnO (3.37 eV), this facilitates the transition of CeO_2_ valence electrons and makes it widely applicable in the field of UV shielding [18,19]. The main challenge in the incorporation of CeO_2_ NPs into polymer matrix is that CeO_2_ NPs are prone to aggregate. Some attempts have been made to prepare CeO_2_ NPs and polymer composites, mainly focusing on modifying the surface of CeO_2_ NPs to improve its dispersion stability in the matrix. However, the process for these methods is complex or complicated conditions are required [20]. Moreover, to the best of our knowledge, only a few researches on the application of CeO_2_ NPs in cellulose-based materials have been reported. By dip-pad-cure or dip-coating processes, cotton fabrics with superhydrophobicity and UV-radiation protection, and silk with UV-shielding ability and antibacterial activity were fabricated respectively [18,21]. Surprisingly, the surface of CeO_2_ NPs was not modified, but its average particle size dispersed in the matrix was nanometer size. However, the optical properties and corresponding mechanisms of CeO_2_–cellulose hybrids have not been investigated in detail as other CeO_2_ hybrids [22]. Moreover, the durability of the performance of the samples prepared by the dip process may be a challenge, requiring a high affinity between CeO_2_ and the fabrics. Recently, by dispersing oxidized cellulose in the hydrothermal system of the CeO_2_ synthesis, nanocomposites with good visible light-induced photocatalytic activity on reduction of aqueous Cr (VI) under acid conditions (pH 4–6) were fabricated [23]. Interestingly, CNs grew on the surface of CeO_2_, accelerating the electron transfer rate of CeO_2_. In these literature examples, cellulose materials were only used as carriers for CeO_2_ NPs or as promoters for promoting CeO_2_ functionality, the characteristics of cellulose-based materials such as transparency and porosity, were not mentioned or utilized.

Herein, with porous RC film as support, transparent nanocomposite films with hydrophobicity and UV-shielding were successfully fabricated by in-situ synthesis of CeO_2_ NPs according to a reported method [24]. Facilitated through interactions between CeO_2_ NPs and cellulose molecules, proved by Fourier transform infrared (FTIR) spectroscopy and X-ray diffraction (XRD) analyses, the uniform dispersion of rod-like CeO_2_ NPs in RC matrix was obtained. Furthermore, the particle size and morphology of in-situ synthesized CeO_2_ NPs were affected by the precursor concentrations and the porous structures of the RC film. Finally, the effects of the concentrations of aq. Ce(NO_3_)_3_·6H_2_O solution on the properties of nanocomposite films such as optical, thermal, and hydrophilic/hydrophobic properties were analyzed. The resulting films with transparency, UV-radiation protection, and hydrophobicity, can be used alone or in combination with other transparent plastic films by hot pressing or calendaring, showing the potential value of application in the field of UV protection.

## 2. Experimental

### 2.1. Materials

Cellulose (cotton linter pulp, α-cellulose >95%) was purchased from Hubei Chemical Fiber Group Ltd. (Xiangfan, China). Cerium nitrate hexahydrate (Ce(NO_3_)_3_·6H_2_O, 99.5%, mass fraction) was provided by the Aladdin reagent company Ltd. (Shanghai, China). Sulfuric acid, urea, and anhydrous sodium sulfate, and other reagents of analytical grade were purchased from Sinopharm Chemical Reagent Co. Ltd. (Shanghai, China) and used without further purification.

### 2.2. Preparation of RC/CeO_2_ Nanocomposite Films

Dissolution of cellulose was conducted according to a reported method [25]. An amount of 5 g of cotton linter pulp was added to a LiOH/urea/H_2_O (8/12/80 in wt%, 100 g) solution and cooled to −20 °C for 24 h. The pre-frozen cellulose solution was vigorously stirred at ambient temperature for 5 min and then subjected to centrifugation at 8000 rpm for 10 min at −4 °C to obtain a transparent cellulose dope. The transparent supernatant fraction was immediately cast on a glass plate, and the resulting gel sheets were immersed into a sulfate aqueous solution to form transparent RC wet films. The wet films were then immersed into aq. Ce(NO_3_)_3_·6H_2_O solution (40 mL) with different concentrations for 10 h at room temperature and atmospheric pressure. The as-obtained films were gently wiped to remove surplus Ce^3+^ ions on the film surfaces, followed by alkaline treatment with 14 mol/L aqueous NaOH solution (40 mL) for 24 h at room temperature, and then were rinsed several times with deionized water.. Finally, the resulting films were dried in air at ambient humidity and pressure at 24 °C, and then treated at 80 °C for 10 h [24]. The as-prepared nanocomposite films with CeO_2_ NPs generated from precursor solutions with concentrations of 0.05, 0.1, 0.2, 0.3 and 0.5 mol/L were denoted as samples RC-0.05, RC-0.1, RC-0.2, RC-0.3, and RC-0.5, respectively.

For comparison, a neat RC film coded as RC was also prepared according to the aforementioned process. Furthermore, pure CeO_2_ NPs were collected outside of the wet film in the aq. Ce(NO_3_)_3_·6H_2_O solution (0.2 mol/L) after in-situ generation of CeO_2_ NPs.

### 2.3. Characterization

The XRD patterns of CeO_2_ powders, RC and RC nanocomposite films were determined on a Bruker D8 Advance Diffractometer (Bruker-AXS, Karlsruhe, Germany) operated in the 2*θ* range of 8–60° with Cu Kα radiation (*λ* = 0.15406 nm) at 40 mV and 40 mA. During the recording of the diffractogram, a narrow slit of 0.1 mm was used with a scanning speed of 0.02/s. The crystallinity χ_c_ (%) of cellulose II was calculated according to the following Equation (1) [26]:

(1) $$CrI=\frac{\left(I_{hkl}-I_{am}\right)}{I_{hkl}}\times 100\%,$$

where *I_hkl_* is the intensity of diffraction maximum of the crystalline regions [200 (21.9°)] in cellulose II; *I_am_* is the intensity value for the amorphous cellulose (around 17.3°). 

Moreover, the crystallite diameter (Dc) of CeO_2_ samples in and outside the RC film were calculated by using the Scherer equation shown in Equation (2) [27]:

(2) D(hkl)=kλ/βCOSθ,

where k is a constant (0.94), *λ* is the wavelength of Cu K*α* radiation (*λ* = 0.15406 nm), *β* is the full-width half-maximum of respective diffraction peak, and 2*θ* is the peak angle in radians.

The samples of CeO_2_ particles, RC, and RC/CeO_2_ nanocomposite films were chemically characterized by attenuated total reflectance infrared (ATR-IR) spectroscopy (Nicolet 560, Nicolet Co., Ltd., Madison, WI, USA). The spectra were recorded from 4000 to 600 cm^−1^ with a resolution of 2 cm^−1^ and a minimum of 16 scans.

The CeO_2_ particles formed outside of the films and the sample of RC-0.2 as a representative of the RC/CeO_2_ nanocomposites were observed using a transmission electron microscope (JEM-2100, JEOL Ltd. Tokyo, Japan) at an accelerating voltage of 200 kV. The surface and cross-section morphologies of RC and RC/CeO_2_ nanocomposite films were measured using a scanning electron microscopy (S-4800, Hitachi Corporation, Tokyo, Japan), with an accelerating voltage of 20 kV, the samples were coated with a thin layer of gold/palladium using a sputter coater (K550X, Emitech Ltd., Kent, UK). The particle size was analyzed by using the software of Nano Measure (Nano Measurer System, version 1.2.5, Fudan University, Shanghai, China). The main constituent elements of sample RC-0.2 were determined by means of energy dispersive spectroscopy (EDS) (attached to the SEM, operating at 20 keV).

Thermal gravimetric analyses of the samples (ca.10 mg) of CeO_2_ particles, RC and RC/CeO_2_ nanocomposite films were carried out by a thermogravimetric analyzer (TGA/SDTA851e, Mettler Toledo instrument Co., Ltd, Zürich, Switzerland) in the temperature range of 30–600 °C under a stream of nitrogen of 50 mL min^−1^ at a heating rate of 10 °C min^−1^.

The ultraviolet absorption properties of CeO_2_ powders, RC, and RC/CeO_2_ nanocomposite films were measured with a UV–Vis near-infrared spectrophotometer (UV-3600 plus, Shimadzu, Kyoto, Japan). The RC nanocomposite films were flattened on the sample plate and the measurement wavelength was 200–800 nm with a barium sulfate coated template selected as a reference. The visible light transmittance of the nanocomposite membranes was measured by a UV–Vis photometer (TU-1901, General Analysis Instrument Co., Ltd., Beijing, China). The thickness of the film samples was about 40 μm.

The contact angle measurements were performed using an OCA 40 dynamic contact angle meter (Data Physics, Stuttgart, Germany). The water contact angle was determined after a water droplet was placed on the film for 60 s. Each static contact angle presented was the average value of those measured at five different locations of each film specimen.

The porosities of the RC and RC/CeO_2_ nanocomposite films were calculated using a reported method [28], the porosity (P) was calculated as follows:

(3) $$P=\frac{\left(M_{1}-M_{2}\right)/q_{1}}{\left(M_{1}-M_{2}\right)/q_{1}+M_{2}/q_{2}}\times 100\%.$$

The wet films were weighed as *M*_1_ and then freeze dried overnight and weighed as *M*_2_. The water content was calculated as *M*_1_ − *M*_2_; *q*_1_ is the water density and *q*_2_ is the RC or RC/CeO_2_ composite density (The densities of the samples were calculated by measuring the weight and volume of the samples).

## 3. Results and Discussion

### 3.1. XRD and FTIR Results

**Figure 1:**
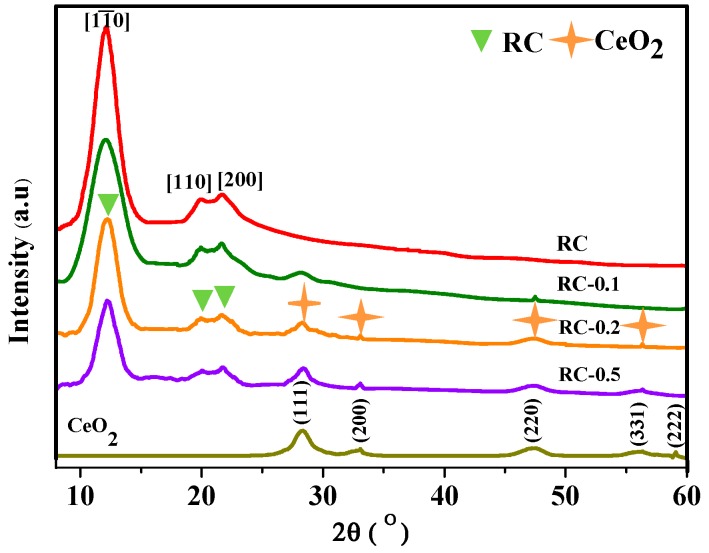
XRD patterns of pure regenerated cellulose (RC) film, CeO_2_ nanoparticles (NPs) as well as cellulose/CeO_2_ nanocomposite films with CeO_2_ NPs generated from Ce(NO_3_)_3_·6H_2_O solutions.

The crystal structures of CeO_2_ NPs, RC, and cellulose/CeO_2_ nanocomposite films were characterized by X-ray diffraction. As shown in Figure 1, the XRD patterns of RC and RC/CeO_2_ nanocomposite films gave peaks at 12.2°, 20.1°, and 21.2°, corresponding to (1$\bar{1}$0), (110), and (200) diffractions of cellulose II, respectively [11]. Moreover, it was evident that the peak intensity of the cellulose II was reduced in the nanocomposite films with increasing concentrations of precursor solution, while the calculated results revealing the crystallinity of RC, RC-0.1, RC-0.2, and RC-0.5 samples were 49.9%, 45.9%, 43.8%, and 42.3%, respectively, showing a decrease in RC crystallinity with the generation of CeO_2_ NPs. Similar results were obtained by other researchers [12]. It can be inferred that the formation and incorporation of CeO_2_ NPs leads to the destruction of the crystallinity of the cellulose matrix to some extent.

In the pattern of CeO_2_ NPs (generated outside of the film in the aq. Ce(NO_3_)_3_·6H_2_O solution with 0.2 mol/L), five diffraction peaks were exhibited at 2*θ* = 28.54°, 33.08°, 47.48°, 56.33°, 59.08°, corresponding to the characteristic (111), (200), (220), (311), and (222) reflections of fluorite phase CeO_2_, respectively (JCPDS No. 34-0394) [29]. No obvious characteristic peaks of the other impurities such as Ce_2_O_3_ or Ce(OH)_3_ were detected. The nanocomposite films also displayed some characteristic peaks of CeO_2_, and the peak intensity of CeO_2_ intensified with increasing precursor concentrations, suggesting an increase in CeO_2_ content in the nanocomposite films. The average crystal sizes of CeO_2_ NPs for CeO_2,_ RC-0.1, RC-0.2, and RC-0.5 sample were 19.2, 13.0, 12.4, and 19.8 nm respectively. Obviously, with porous RC film as supporting medium, at the same precursor concentration (CeO_2_ NPs vs. RC-0.2), the interactions between porous cellulose films and CeO_2_ NPs prevented the growth of larger crystals. Moreover, the calculated results revealed that with porous RC film as support, the precursor concentration had little influence on the crystalline size of in-situ synthesized CeO_2_ NPs except at high Ce^3+^ concentrations. At low Ce^3+^ ions concentrations, due to the electron-rich oxygen atoms of polar hydroxyl and ether groups of the cellulose macromolecule, predictable interaction between the porous RC film and electropositive Ce^3+^ ions would prevent the growth of larger crystals [30]. However, when the concentration of Ce^3+^ ions was so high that the porous cellulose had difficulty to exert effective electrostatic action on excessive metal ions, its control effect on the crystal size was weakened.

**Figure 2:**
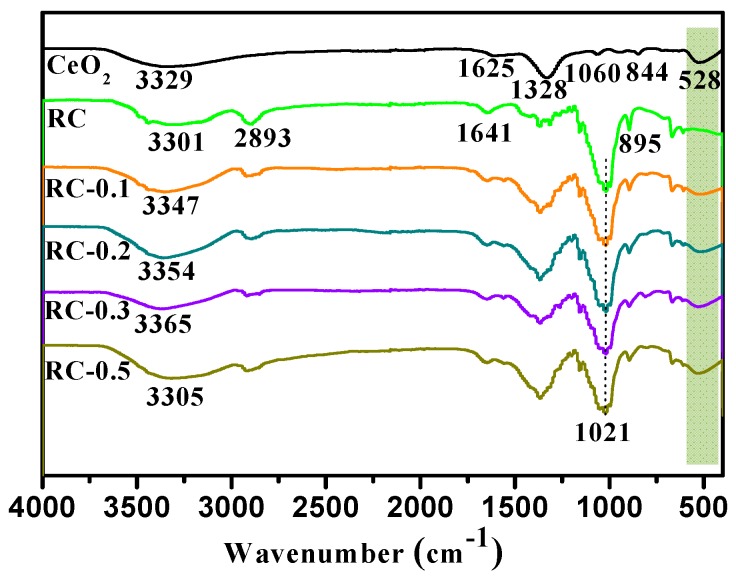
FTIR spectra of CeO_2_ particles, RC and RC/CeO_2_ nanocomposite films.

From Figure 2, it is clearly evident that as-received RC and RC/CeO_2_ film samples showed absorption peaks of cellulose II at around 3350, 2893, 1641, 1021, and 895 cm^−1^, attributed to the OH, CH_2,_ crystallization water, C–O, and stretching vibration of C1 respectively, indicating the complete conversion of cellulose I to cellulose II after alkaline treatment [31]. As shown in Figure 2, in the spectrum of CeO_2_ NPs, the absorption bands at 3329, 1625, 1328, 1060, and 844 cm^−1^, were attributed to the stretching mode of water and hydroxyl groups and the vibrations associated with the incoordination of the adsorbed NO_3_^−1^ ions respectively [32,33]. In addition, the peak at 528 cm^−1^, corresponding to Ce–O stretching [20], could be observed in the spectra of the CeO_2_, and nanocomposite films, indicating the successful in-situ synthesis of CeO_2_. Furthermore the stretching vibration bands of the hydroxyl groups of cellulose at 3300–3650 cm^−1^ was shifted to a higher wavenumber with increasing Ce^3+^ concentration form 0 to 0.3 mol/L, suggesting a reduction in hydrogen bonding between cellulose molecules and an increased interaction between the hydroxyl group of cellulose and CeO_2_ NPs [30]. However, as the Ce^3+^ concentration reached 0.5 mol/L, the OH peak shifted to a lower wavenumber, indicating a reduction of the interaction between CeO_2_ NPs and RC, due to aggregation of CeO_2_ NPs, as shown in SEM (Figure 4).

### 3.2. Morphology and Structure of Nanocomposite Films

**Figure 3:**
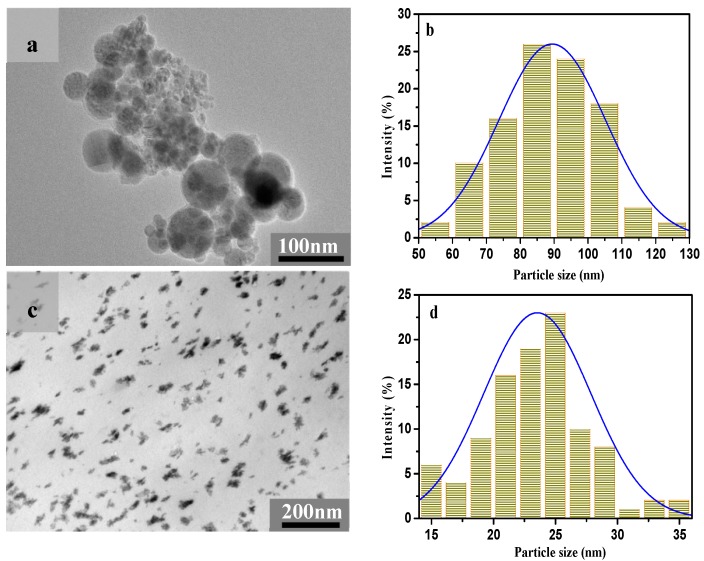
(**a**) TEM image of CeO_2_ NPs formed outside the film; (**b**) particle size histograms of CeO_2_ NPs corresponding to (a); (**c**) TEM image of the cross-section of cellulose/CeO_2_ nanocomposite films; (**d**) particle size histogram of CeO_2_ NPs estimated from (**c**). (Two samples were generated form 0.2 mol/L aq. Ce(NO_3_)_3_·6H_2_O solution).

It is obvious that the CeO_2_ NPs formed outside of the film using 0.2 mol/L aq. Ce(NO_3_)_3_·6H_2_O solution were spherical in shape (Figure 3a). Figure 3b represents the corresponding particle size distribution of CeO_2_ NPs. It is evident that the diameter of the CeO_2_ NPs varied from 50 to 130 nm with an average value of 75 ± 8 nm. For comparison, the TEM image of the cross-section of the nanocomposite film with CeO_2_ NPs generated using the same concentration of aq. Ce(NO_3_)_3_·6H_2_O is presented (Figure 3c). Obviously, CeO_2_ NPs exhibited flake-like morphology with irregular shapes and with average particle size of 24 ± 3 nm. Moreover, CeO_2_ NPs were dispersed uniformly in the cellulose matrix. These results meant that the micro and nanoporous structure of RC films supplied not only nanoreacting sites for the formation of the CeO_2_ NPs, but also a shell to protect their nanostructure. The irregular shape of CeO_2_ NPs implied CeO_2_ NPs could freely rotate, and randomly align within the pores of the RC film [11].

**Figure 4:**
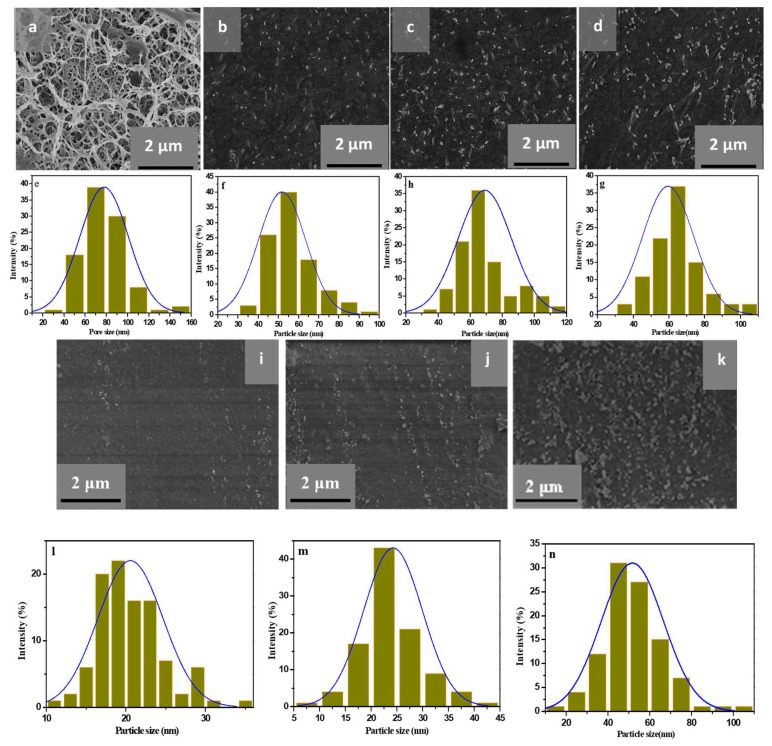
SEM image (**a**) and particle size histogram (**e**) of the surface of RC; SEM images and particle size histograms of the surface of RC-0.1 (**b**,**f**); RC-0.2 (**c**,**g**); RC-0.5 (**d**,**h**) and the cross-section of RC-0.1 (**i**,**l**); RC-0.2 (**j**,**m**); RC-0.5 (**k**,**n**).

To investigate the effects of precursor concentrations and porous structures of RC film on the morphologies of CeO_2_ NPs, Figure 4 shows the SEM images of surface (a–d) and cross-section (i–k) of the RC and RC/CeO_2_ nanocomposite films with CeO_2_ NPs generated using 0.1, 0.2, and 0.5 mol/L aq. Ce(NO_3_)_3_·6H_2_O solutions, respectively. Correspondingly, the pore size of the RC film ((surface, e) and particle size histograms of CeO_2_ NPs (surface, f–h; cross-section, l–n) were also presented (Figure 4). It was evident that the RC film (Figure 4a) represented a homogeneous porous structure (average pore size, 80 ± 19 nm), as a result of the phase separation of the cellulose solution during the regenerating process. Thus, as the RC films were immersed into Ce(NO_3_)_3_·6H_2_O solutions, Ce^3+^ could be readily impregnated into the cellulose films through the pores, and then the Ce^3+^ ions could bind to cellulose fibers via electrostatic interaction, facilitated through the negative charge on the surface of the cellulosic material due to ionization of hydroxyl groups when immersed in water [34]. Then, the RC film would provide nanoreacting sites for in situ synthesis of CeO_2_ NPs. Correspondingly, a significant change in pore structure of RC films with generated CeO_2_ NPs would occur. The calculated porosity of RC, RC-0.1, RC-0.2, and RC-0.5 film were 91%, 50%, 43%, and 30%, respectively, as a result of filling the porous structure of the cellulose film with CeO_2_ NPs.

From Figure 4, it is clearly evident that the CeO_2_ NPs formed with RC film as support were fairly uniformly distributed in the nanocomposites. However with increasing precursor concentrations the agglomeration of CeO_2_ NPs in the nanocomposite films became obvious, due to increased CeO_2_ NPs content. This was consistent with reduced transparency of the nanocomposite films and gradual yellowing of the nanocomposite films as the precursor concentrations increased (Figure 6c). Furthermore, the surface roughness of the nanocomposite films increased with increasing precursor concentrations. In the case of the size distribution of CeO_2_ NPs, the average widths of the nanoparticles observed in the slice parallel to the surface of the films, were 51 ± 4 nm for RC-0.1, 60 ± 6 nm for RC-0.2, and 71 ± 12 nm for RC-0.5, respectively. While the slice was perpendicular to the plane of nanocomposite films, the average diameters of nanoparticles were 21 ± 5 nm for RC-0.1, 25 ± 6 nm for RC-0.2, and 51 ± 10 nm for RC-0.5, respectively. It could be concluded that the CeO_2_ NPs were rod-like shape comparing the size of CeO_2_ particles parallel to the surface of the film and the vertical direction in the SEM images. Though in situ generation of CeO_2_ NPs with RC film as supporting medium could avoid agglomeration as proved by TEM analyses, it could be found that the size of the CeO_2_ NPs increased with increasing precursor concentrations in the SEM images. Especially, when 0.5 mol/L aq. Ce(NO_3_)_3_·6H_2_O was used, agglomeration could not be avoided. Within a certain range of precursor concentration, due to the electrostatic action, at higher Ce^3+^ ions concentrations, larger amounts of Ce^3+^ ions were adsorbed on the cellulose fibers, leading to a higher number of CeO_2_ NPs generated which in turn increased the particle size. However, with a further increase of Ce^3+^ ion concentrations, agglomeration would occur due to the interaction between cellulose and CeO_2_ NPs, which results in difficulty in overcoming the aggregation effect of the INPs in order to reduce the surface energy.

**Figure 5:**
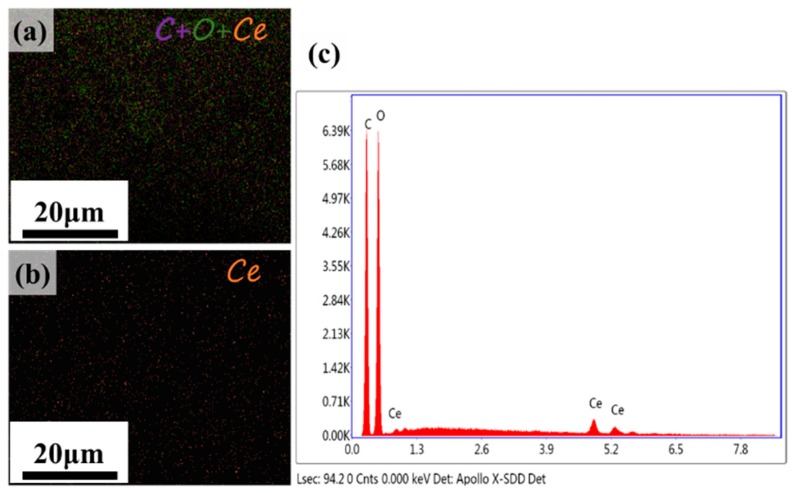
(**a**) C + O + Ce; (**b**) Ce EDS mapping images and (**c**) EDS analysis of the surface of the RC-0.2 film.

Moreover, energy dispersive spectrum (EDS) (Figure 5) from SEM indicated that there were only C, O and Ce elements in the nanocomposite film, which further confirmed the CeO_2_ NPs were effectively synthesized in the RC films.

### 3.3. Optical Properties of Nanocomposite Films

**Figure 6:**
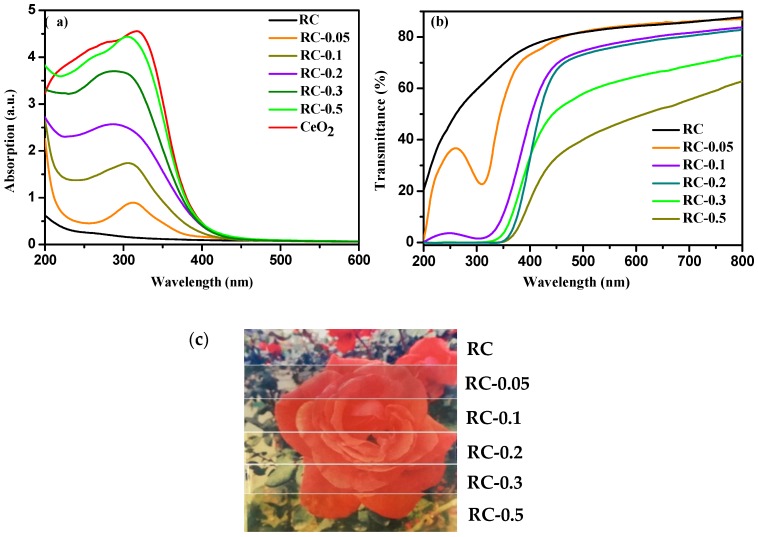
(**a**) Ultraviolet absorption property of CeO_2_ powders, RC, and RC/CeO_2_ nanocomposite films (**b**) UV–Vis transmittance of RC and RC/CeO_2_ nanocomposite films; (**c**) digital pictures of RC and RC/CeO_2_ nanocomposite films (tested film with a thickness of approximately 40 μm).

Figure 6a shows the UV–Vis absorption spectra of RC and RC/CeO_2_ nanocomposite films, and CeO_2_ powders. It was evident that the RC film exhibited poor absorption at a wavelength ranging from 200 to 800 nm, while CeO_2_ showed a strong absorption peak in the UV range and had no absorption band above 500 nm, resulting from the wide band gap and the strong scattering effect of the CeO_2_ NPs. Interestingly, the RC/CeO_2_ nanocomposite films possessed intense absorption in the UV range, especially and presented good absorption in the UV-A (320–400 nm) and UV-B (280–320 nm) region, indicating that the organic–inorganic nanocomposites retained the inherent optical properties of the CeO_2_ NPs and could be used as efficient UV absorber materials in some fields. Furthermore, the absorption edge was beyond 400 nm, indicating the light yellow of the nanocomposite films [35], and with increasing precursor concentrations, this trend became obvious (Figure 6c). Meanwhile, an increase in the intensity and width of the band corresponding to the UV absorption peak was observed, suggesting more CeO_2_ NPs were generated in situ. 

The transmittance of the RC/CeO_2_ nanocomposite films in the visible light region (400–800 nm) decreased with increasing precursor concentrations (Figure 6b), mainly attributed to larger CeO_2_ NPs generated and some agglomeration of CeO_2_ NPs, as revealed by XRD and SEM analyses. Moreover, the immobilization of CeO_2_ NPs into RC nanocomposite films led to the decrease in transparency of the nanocomposite films in the UV region (200–400 nm), due to the high UV absorption of CeO_2_ NPs. This is particularly true at high precursor concentrations. From Figure 6c, it can be seen that, obviously, all the RC nanocomposite films showed good transparency. However, as the precursor concentrations increased, the yellowing of the film became obvious, attributable to the yellowing of CeO_2_ particles.

**Figure 7:**
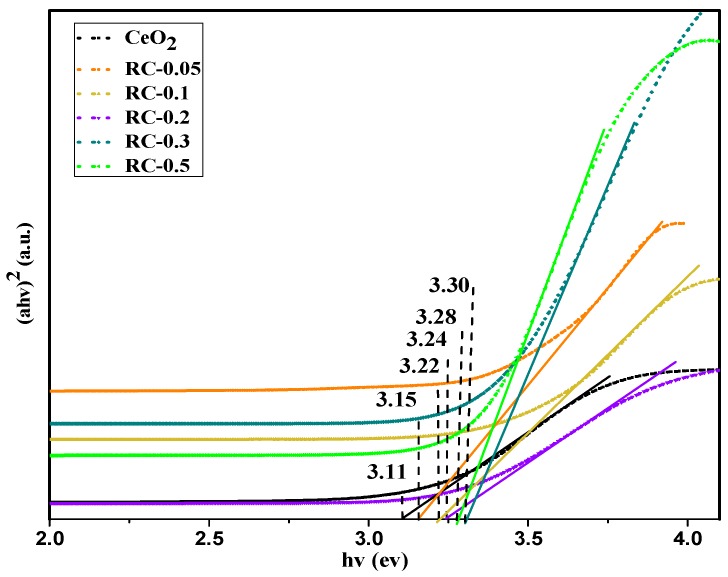
Plot of (αhv)^2^ versus photon energy for the CeO_2_ NPs and CeO_2_ NPs dispersed in the RC matrix.

The band gap energy *E_g_* for the CeO_2_ NPs could be determined using the following equation [35]:

(4) $${\left(\alpha h\nu \right)}^{2}=k\left(h\nu -E_{g}\right),$$

where *α* is the absorption coefficient, and can be calculated according to the following equation: $\alpha =\frac{2.303\times 10^{3}A\rho }{LC}$, where *A* is the absorbance of the sample, *ρ* is the real density of CeO_2_ (7.172 g cm^−3^), *L* is the path length of the nanocomposite film, and *C* is the loading of the nanoparticles in the nanocomposite, k is the parameter, h*υ* is the absorption energy, and *E_g_* is the band gap energy [36]. The optical band gap was estimated by extrapolating the straight-line region in the plot of (*α*h*υ*)^2^ versus photon energy (Figure 7).

According to previous researches, as a result of the quantum confinement effect, the value of blue-shifting resulting from the reduction of particle size, is inversely proportional to the square of the particle size [37]. Wherefore as shown in the spectra, corresponding to the change in the size of CeO_2_ NPs, in comparison to pure CeO_2_ NPs, the absorption for all the nanocomposite samples was blue shifted. However, on increasing the precursor concentrations, a clear blue-shift of the absorption could be observed, and then a red-shift. It was evident that the CeO_2_ NPs generated with RC film as support led to an increase in the *E_g_* values, compared to pure CeO_2_ (3.11 eV). This was attributed to the control of the particle size and the morphology of CeO_2_ NPs by the porous RC film. 

Furthermore, the *E_g_* value of CeO_2_ hybrids increased with the increase of the precursor concentration from 0.05 to 0.3 mol/L. Theoretically, the absorption of ceria in the UV region originates from the charge–transfer transition between the O 2p and Ce 4f states in O^2−^ and Ce^4+^, and this absorption is much stronger than the 4f^1^–5d^1^ transition from the Ce^3+^ species [38]. Usually, with the increasing amount of CeO_2_, the Ce^3+^ concentrations of total Ce decrease [39]. As a result, the reduction of Ce^3+^ concentration of total Ce leads to an increase in *E_g_* [40]. However, the *E_g_* values decreased slightly with further increase of the precursor concentrations, due to the agglomeration of nanoparticles, attributed to the surface effect originating from the indirect quantum size [41].

### 3.4. Thermal Stability Properties of the RC and Nanocomposite Films

**Figure 8:**
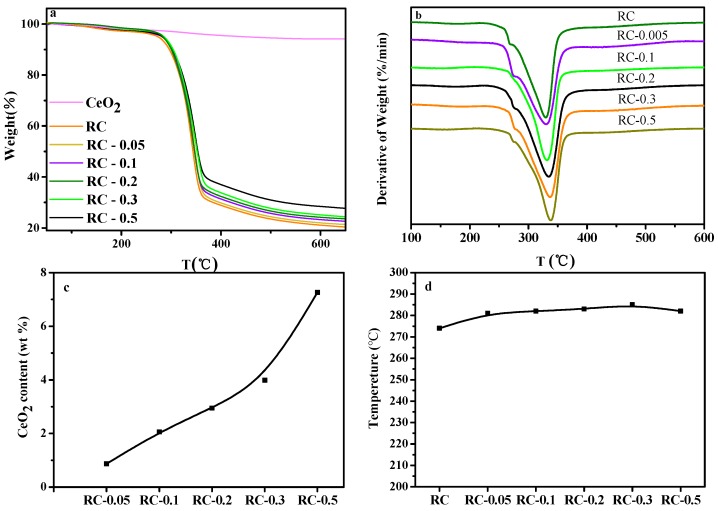
TGA (**a**) and DTG (**b**) curves for RC and RC/CeO_2_ nanocomposite films under nitrogen atmosphere; (**c**) the influences of Ce(NO_3_)_3_·6H_2_O concentrations on the content of the incorporated CeO_2_ NPs (wt%) in the nanocomposite films; (**d**) the temperature at 5 wt% decomposition of RC and RC/CeO_2_ nanocomposite films.

Figure 8a,b shows the TG and DTG curves of the RC and RC/CeO_2_ nanocomposite films. Obviously, RC/CeO_2_ composite films had slightly better thermal stability at the initial stage of material degradation (Figure 8d). Because the initial thermal weight loss of cellulose-based materials mainly resulted from evaporation of moisture [11], this phenomenon could thus be attributed to the interactions between CeO_2_ NPs and cellulose molecules that reduce the amount of free hydroxyl groups and the hydrophobic properties of the CeO_2_ NPs [42]. The temperature at 5 wt% decomposition of the RC-0.5 sample was lower than that of the other composite membranes, mainly due to the agglomeration of CeO_2_ NPs, resulting in a reduction in the interaction between CeO_2_ NPs and cellulose, as revealed by FTIR. In general, all the sample films had thermal degradation at 230–370 °C, irrespective of the CeO_2_ NPs content, indicating that the presence of CeO_2_ NPs had almost no effect on the thermal degradation behavior of RC. However, the introduction of CeO_2_ NPs resulted in a slight increase in the maximum thermal decomposition temperature of the nanocomposite films (Figure 8b), resulting from the interaction between CeO_2_ NPs and cellulose molecules. Moreover, higher concentrations of aq. Ce(NO_3_)_3_·6H_2_O led to higher content of CeO_2_ NPs in as-prepared RC nanocomposite films (Figure 8c), as evidenced by the relevant SEM and XRD analyses.

### 3.5. Surface Hydrophilic and Hydrophobic Properties of RC Nanocomposite Films

**Figure 9:**
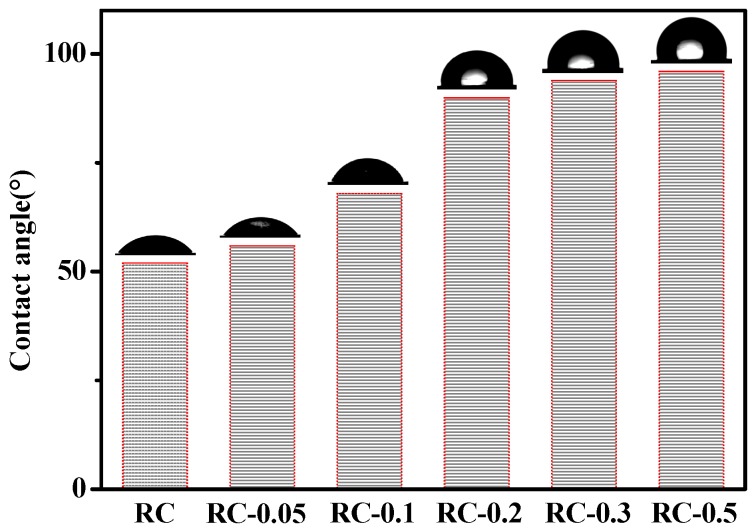
Static contact angle of the RC and RC/CeO_2_ nanocomposite films.

The water contact angles on RC and nanocomposite film surfaces were recorded (Figure 9). The hydrophilic nature of the RC films was well demonstrated by a low water contact angle of 52°. Interestingly, as CeO_2_ NPs were introduced into the RC films, the contact angle values increased to 90°, 94°, and 96° for the RC-0.2, RC-0.3, and RC-0.5 films, respectively. This was mainly due to the increase in surface roughness of the composite film as the CeO_2_ NPs content increased from 0 wt% to 7.3 wt%, as revealed by SEM images. Similar conclusions were obtained in the study of ZnO/TEMPO oxidized cellulose nanofibril composite films. In their study, when the content of ZnO reached 10 wt%, the contact angle (95°) and the surface roughness of the composite film reached the maximum at the same time [42]. Furthermore, the hydrogen bond interaction between CeO_2_ NPs and cellulose proved by FTIR, reduces the free hydroxyl in the system and this promotes the hydrophobicity of the composite films. When the CeO_2_ NPs content increased to a certain value, the water contact angle of the composite film increased slowly, indicating that the interaction between CeO_2_ NPs and cellulose decreased due to the agglomeration of CeO_2_ NPs. The hydrophobicity of the CeO_2_ NPs themselves may be another reason for the increased hydrophobicity of the composite membrane. Azimi et al. attributed this hydrophobicity of rare-earth oxides to their unique electronic structure, where the unfilled 4f orbitals are shielded from interactions with the surrounding environment by the full octet of electrons in the 5s^2^p^6^ outer shell. Consequently, these metal atoms would have a lower tendency to exchange electrons and form a hydrogen bond with interfacial water molecules [43].

## 4. Conclusions

In summary, a direct and facile synthetic strategy was provided to successfully incorporate nanodispersed CeO_2_ particles in RC films, thereby affording UV-shielding nanocomposite films. The porous RC film with a lot of hydroxyl groups can interact with electropositive transition-metal cations and act as effective nanoreactors for in situ synthesis of metal nanoparticles and thereby the function of RC film can be realized. With porous RC film as supporting medium, the morphology and particle size of the CeO_2_ NPs and accordingly the properties of as-prepared nanocomposite films were affected by the concentrations of the precursor and the porous structure of RC film. As-prepared RC/CeO_2_ nanocomposite films by in situ synthesis with the appropriate precursor concentration, exhibited moderate thermal stability, a certain degree of hydrophobicity, high transmittance and the desired UV shielding properties. These end products show potential applications in areas such as optical functional materials.
